# Percutaneous Coronary Intervention Facility Access and Clinical Outcomes Among Patients With Acute Myocardial Infarction

**DOI:** 10.1001/jamanetworkopen.2026.38687

**Published:** 2026-10-12

**Authors:** Yu-Chu Shen, Renee Y. Hsia

**Affiliations:** 1 Department of Acquisition, Finance, and Manpower, Naval Postgraduate School, Monterey, California; 2 National Bureau of Economic Research, Cambridge, Massachusetts; 3 Department of Emergency Medicine, University of California, San Francisco; 4 Philip R. Lee Institute for Health Policy Studies, University of California, San Francisco

## Abstract

**Importance:**

The proliferation of percutaneous coronary intervention (PCI) facilities across the US has raised questions about whether expanding capacity improves outcomes in areas already served vs those without prior access.

**Objective:**

To evaluate the association between the opening of PCI-capable facilities and outcomes following acute myocardial infarction (AMI) in communities with and without baseline PCI access.

**Design, Setting, and Participants:**

This cohort study used California data from inpatient, emergency department, and ambulatory surgery encounters from January 1, 2011, to December 31, 2022, including all adults hospitalized with a primary diagnosis of AMI. Zip code communities were categorized by baseline (2011) access to PCI-capable facilities within a 30-minute drive.

**Exposure:**

Opening of a PCI-capable facility within 30 minutes of a community’s zip code during the study period.

**Main Outcomes and Measures:**

Primary outcomes were (1) admission to a PCI-capable facility, (2) receipt of PCI during the index hospitalization, (3) unscheduled acute care visit within 30 days, and (4) all-cause mortality at 30 days and 1 year. Linear probability models with community and time-fixed effects estimated changes in outcomes following PCI facility openings separately for communities with and without baseline access.

**Results:**

Among 590 282 patients with AMI (157 435 younger than age 60 years [27%]; 222 773 female [38%]; 63 460 Asian [11%], 129 137 Hispanic [22%], 315 326 White [53%]), 500 778 (85%) lived in communities with baseline PCI access, and 89 504 (15%) lived in communities without; 18 805 patients (21%) without baseline access experienced a PCI opening. Openings in communities without baseline access were associated with a 30.0 percentage point (pp) increase (95% CI, 28.2 to 31.8 pp) in admission to PCI-capable hospitals and a 5.1 pp increase (95% CI, 3.3 to 7.0 pp) in PCI receipt, along with reductions in 30-day (–1.8 pp; 95% CI, –3.2 to –0.5 pp) and 1-year (–2.8 pp; 95% CI, –4.7 to –1.0 pp) mortality. In contrast, openings in communities with baseline access were associated with smaller gains in admission (1.5 pp; 95% CI, 1.1 to 1.9 pp), modest increases in PCI use (1.0 pp; 95% CI, 0.5 to 1.6 pp), decreases in unscheduled visits within 30 days (–0.7 pp; 95% CI, –1.2 to –0.2 pp), and no mortality change. Sensitivity analyses restricted to higher-volume PCI centers (50 annual cases or more) yielded consistent findings.

**Conclusions and Relevance:**

In this cohort study of 590 282 patients with AMI in California, opening new PCI facilities was associated with substantial improvements in communities without prior PCI availability, including a 53% relative increase in access to admission, 16% increase in PCI use, and 20% and 15% reductions in 30-day and 1-year mortality, respectively. In contrast, already served areas showed smaller benefits and no mortality improvement. PCI expansion should prioritize underserved regions to maximize population-level benefits and avoid inefficient duplication of services.

## Key Points

**Question:**

Is the opening of new percutaneous coronary intervention (PCI) facilities associated with improved access, treatment, and survival for patients with acute myocardial infarction (AMI), and does this association differ between communities with and without baseline PCI access?

**Findings:**

In this cohort study of 590 282 AMI hospitalizations in California from 2011 to 2022, openings of PCI facilities were associated with significant increases in access to PCI-capable hospitals, higher rates of PCI treatment, and reductions in 30-day and 1-year mortality in communities without baseline PCI access. In contrast, openings in communities with existing PCI capacity were associated with small improvements in access and treatment but no significant mortality benefit.

**Meaning:**

Expansion of PCI services was associated with improved outcomes in previously underserved communities but provided limited benefit in areas already served, suggesting that future health system and policy decisions should prioritize underserved regions to maximize population health benefit and avoid unnecessary duplication of high-cost cardiac services.

## Teaser

This cohort study of hospitalizations for acute myocardial infarction in California examines the opening of new percutaneous coronary intervention facilities with treatment access and mortality.

## Introduction

Percutaneous coronary intervention (PCI) is among the most rigorously validated and definitive therapies for acute myocardial infarction (AMI), with extensive evidence demonstrating improved survival and reduced complications when performed promptly.^1,2^ Over the past 2 decades, the US has experienced tremendous growth in PCI facilities^3,4,5^ driven by clinical guidelines, policies, and hospitals’ interest in expanding specialized services.^6,7,8^ While greater availability may facilitate treatment for patients with ST-elevation myocardial infarction (STEMI) and many with non–ST-elevation MI (NSTEMI), duplication of services may fragment care, reduce procedural volume at individual centers, and compromise quality as a result.^4,9,10,11^ Prior studies have demonstrated that volume affects outcomes, with higher PCI volumes associated with lower mortality.^12,13^

Despite broad consensus regarding the benefits of PCI for AMI, it remains unclear whether opening new PCI centers in communities with existing PCI facilities provides incremental benefits or instead dilutes expertise and resources. Conversely, for communities without baseline access, new PCI facilities may reduce geographic barriers, increase the likelihood of timely treatment, and improve survival. Evidence is limited regarding how these differing baseline contexts shape patient access, treatment patterns, and post-AMI outcomes.

Given these uncertainties, rigorous evaluation of PCI expansion is needed to guide policy and health system planning. While expansion has likely improved access to care in many areas,^4,14^ it also raises important questions about how PCI is being utilized, particularly which patients are most likely to receive it. Therefore, we examined the association between the opening of PCI facilities and AMI patient outcomes in communities with and without baseline PCI access between 2011 and 2022. We hypothesized that new PCI facilities would be associated with greater improvements in access, PCI use, and survival in communities without baseline access, but that benefits in communities with existing PCI capacity would be smaller and possibly mixed.

## Methods

This study was determined to be nonhuman participants research by University of California, San Francisco, and so did not require informed consent. We followed the Strengthening the Reporting of Observational Studies in Epidemiology (STROBE) reporting guideline for cohort studies.

### Study Design and Data Sources

We used patient-level data from 3 nonpublic datasets maintained by the California Department of Health Care Access and Information (HCAI): the Patient Discharge Data, the Emergency Department Data, and the Ambulatory Surgery Data. Together, these sources capture both inpatient and outpatient encounters and enable comprehensive identification of patients with AMI in California. All 3 datasets include unique patient record identifiers to link records for follow-up visits and mortality, information on patient demographics (age, sex, self-reported race and ethnicity), residential zip code, primary and secondary diagnoses, procedure codes and dates, insurance coverage, planned or unscheduled hospital admissions, comorbid conditions, and facility identifier to link to hospital-level information.

We obtained hospital-level information from HCAI’s annual facility utilization files, which report PCI procedure volumes, ownership type, and facility characteristics. These files also provide geographic coordinates for each hospital. To measure geographic access to PCI hospitals from each patient’s zip code community, we combined hospital geolocations with US Census 2010 zip code centroid data and used web-based mapping applications to obtain driving times from each community centroid to PCI-capable facilities under routine traffic conditions. This drive-time database enables us to determine whether a community has access to PCI-capable facilities within a 30-minute drive time. Lastly, we identified rural communities from the Zip Code Approximation file from the Federal Office of Rural Health Policy under the Health Resources & Services Administration.^15^

### Study Population

The study population consisted of all patients residing in California with a primary diagnosis of AMI between January 1, 2011, and December 31, 2022, as classified using International Classification of Diseases (ICD) diagnosis codes, specifically *International Classification of Diseases, Ninth Revision, Clinical Modification (ICD-9-CM) *prior to October 2015 and *International Statistical Classification of Diseases, Tenth Revision, Clinical Modification (ICD-10-CM)* thereafter. We included patients with both STEMI and NSTEMI, because early invasive management is also guideline-recommended for selected patients with NSTEMI (eg, persistent symptoms, dynamic ischemic electrocardiogram [ECG] changes, high GRACE [Global Registry of Acute Coronary Events] score).^16,17^ Moreover, decisions to open PCI facilities affect care delivery for all patients with AMI, not solely those with STEMI. Excluding NSTEMI would therefore underestimate the broader system-level impact of PCI expansion on access, treatment patterns, and outcomes. We identified PCI procedures using procedure codes (list provided in eTable 1 in Supplement 1). We excluded patients with missing record identifiers because we could not link their records across all patient databases to capture their complete medical history and postdischarge outcomes, and also patients missing geographic identifiers and those residing more than 100 miles from the treating facility, on the assumption that these cases likely represented individuals who received PCI while away from their usual place of residence.

### Exposure

The primary exposure was the opening of a PCI-capable facility within a 30-minute driving time of the patient’s zip code community during the study period. PCI facilities were defined as facilities that performed at least 10 PCIs per year.

### Outcomes

We evaluated 4 patient outcomes: (1) admission to a PCI-capable facility during the index hospitalization; (2) receipt of PCI during the index hospitalization; (3) unscheduled acute care visit (ie, emergency department [ED] visit or readmission) within 30 days of index admission, which is commonly used in previous studies as a measure of postdischarge care coordination and fragmentation; and (4) 30-day and 1-year all-cause mortality. Due to changes in HCAI data collection process, an unscheduled acute care visit occurred if any one of the following conditions were met within the 30-day window from the index admission: (1) the subsequent record was from the ED discharge, (2) if the admission type was unscheduled on or before 2017, or (3) if the admission type was urgent or emergency after 2017.

### Statistical Analysis

We implemented linear probability models with community- and time-fixed effects to estimate the association between the opening of PCI facilities and patient outcomes. Models adjusted for demographics (eg, age, sex, race and ethnicity, insurance), clinical factors (eg, comorbidities using standard Elixhauser codes,^18^ including diabetes, vascular disease, heart failure, kidney disease, stroke, and prior AMI or coronary artery bypass graft within 12 months), and year indicators. Race and ethnicity categories were included as covariates in the model because prior work has shown systematic differences in care-seeking behavior and health outcomes across race and ethnicity, and included Asian or Pacific Islander, Black, Hispanic, Native American, White, and other (unknown, multiple races, invalid, and self-identifying as other). We used linear probability models to estimate associations in the presence of fixed effects. The community fixed effects were critical because they removed time-invariant unobserved differences across communities, like underlying differences in general population health, culture, and socioeconomic conditions. The time-fixed effects control for macro trends that affect all communities, such as the transition from *ICD-9* to *ICD-10* coding in October 2015 and the economic shock of the pandemic in 2020. In the linear probability models, coefficients were expressed as percentage-point (pp) changes in outcomes, representing differences in outcomes before and after a community was exposed to PCI facility openings, relative to changes in outcomes in the reference communities, those with no exposure to newly opened PCI facilities, during the same period. Robust standard errors were clustered at the community level. We estimated the models separately for 2 types of communities: (1) communities that already had PCI-capable hospitals within a 30-minute drive time at baseline (2011), and (2) communities without such capacity at baseline.

We performed several sensitivity analyses, such as restricting the exposure to higher-volume PCI centers (50 annual cases or more) and stratifying the analysis by patients’ health conditions. Analyses were conducted in Stata version 19 (StataCorp). Statistical significance was defined as 2-sided *P* < .05. Analysis was completed between May 2025 and July 2026.

## Results

From 2011 through 2022, we identified 590 282 patients hospitalized with AMI in California communities (157 435 younger than age 60 years [27%]; 222 773 female [38%]; 63 460 Asian [11%], 129 137 Hispanic [22%], 315 326 White [53%]) (Table). Of these, 500 778 patients (85%) resided in communities with baseline PCI access, and 89 504 (15%) resided in communities without baseline PCI access. Figure 1 shows the geographic distribution of the 2 types of communities and the opening years and locations of PCI facilities in California during our study period. Among patients with no PCI access at baseline, 42 584 (48%) were from rural communities; 18 805 patients (21%) in communities without baseline access to PCI facilities experienced the opening of such facilities between 2012 and 2022 (Table). In contrast, 397 960 patients (79%) in communities that already had PCI facilities nearby at baseline had additional PCI facilities open within a 30-minute drive time.

**Table.** Descriptive Statistics of Patients Acute Myocardial Infarction (AMI) by Baseline Access

Characteristics	Patients, No. (%)		
	Whole sample (N = 590 282)	No PCI within 30 min at baseline (n = 89 504)	Had PCI within 30 min at baseline (n = 500 778)
Community			
Rural	52 756 (9)	42 584 (48)	10 172 (2)
Experienced opening during study period	416 765 (71)	18 805 (21)	397 960 (79)
Race and ethnicity			
Asian or Pacific Islander	63 460 (11)	2502 (3)	60 958 (12)
Black	47 988 (8)	3038 (3)	44 950 (9)
Hispanic	129 137 (22)	18 002 (20)	111 135 (22)
Native American	2165 (<1)	783 (1)	1382 (<1)
White	315 326 (53)	60 995 (68)	254 331 (51)
Other races^a^	32 206 (5)	4184 (5)	28 022 (6)
Sex			
Female	222 773 (38)	34 137 (38)	188 636 (38)
Male	367 493 (62)	55 353 (62)	312 140 (62)
Unknown or nonbinary sex	16 (<1)	14 (<1)	2 (<1)
Age distribution at time of admission			
0-59 y	157 435 (27)	22 870 (26)	134 565 (27)
60-64 y	74 026 (13)	11 637 (13)	62 389 (12)
65-69 y	77 282 (13)	12 466 (14)	64 816 (13)
70-74 y	73 701 (12)	12 139 (14)	61 562 (12)
75-79 y	67 579 (11)	10 595 (12)	56 984 (11)
80-84 y	59 302 (10)	8972 (10)	50 330 (10)
≥85 y	80 957 (14)	10 825 (12)	70 132 (14)
Insurance coverage			
Medicare	347 755 (59)	55 418 (62)	292 337 (58)
Medicaid or indigent	79 027 (13)	12 038 (13)	66 989 (13)
Private	139 137 (24)	17 522 (20)	121 615 (24)
Self-pay	12 887 (2)	2034 (2)	10 853 (2)
Other	11 476 (2)	2492 (3)	8984 (2)
Clinical characteristics			
STEMI	138 603 (23)	20 541 (23)	118 062 (24)
NSTEMI	451 679 (77)	68 963 (77)	382 716 (76)
Peripheral vascular disease	84 804 (14)	9092 (10)	75 712 (15)
Pulmonary circulation disorders	19 289 (3)	2771 (3)	16 518 (3)
Diabetes	222 070 (38)	30 606 (34)	191 464 (38)
Kidney failure	157 919 (27)	18 739 (21)	139 180 (28)
Liver disease	17 686 (3)	2216 (2)	15 470 (3)
Cancer	16 627 (3)	2231 (2)	14 396 (3)
Dementia	26 486 (4)	3304 (4)	23 182 (5)
Valvular disease	68 369 (12)	9232 (10)	59 137 (12)
Hypertension	463 507 (79)	66 163 (74)	397 344 (79)
Chronic pulmonary disease	111 409 (19)	18 222 (20)	93 187 (19)
Rheumatoid arthritis or collagen vascular	12 900 (2)	1799 (2)	11 101 (2)
Coagulation deficiency	39 366 (7)	4443 (5)	34 923 (7)
Obesity	103 792 (18)	14 089 (16)	89 703 (18)
Alcohol abuse	22 012 (4)	3676 (4)	18 336 (4)
Drug abuse	22 390 (4)	3402 (4)	18 988 (4)
Depression	32 115 (5)	4813 (5)	27 302 (5)
Psychosis	41 599 (7)	5016 (6)	36 583 (7)
Hypothyroidism	67 072 (11)	9721 (11)	57 351 (11)
Paralysis and other neurological disorder	43 286 (7)	5711 (6)	37 575 (8)
Ulcer	3078 (1)	419 (<1)	2659 (1)
Weight loss	23 444 (4)	2850 (3)	20 594 (4)
Fluid and electrolyte disorders	135 987 (23)	18 319 (20)	117 668 (23)
Anemia (blood loss and deficiency)	121 007 (20)	13 749 (15)	107 258 (21)
Atrial flutter	118 397 (20)	16 876 (19)	101 521 (20)
Heart failure	226 644 (38)	31 536 (35)	195 108 (39)
Chronic kidney disease	167 186 (28)	19 583 (22)	147 603 (29)
Hyperlipidemia	374 507 (63)	48 339 (54)	326 168 (65)
Stroke	24 949 (4)	3187 (4)	21 762 (4)

Abbreviations: PCI, percutaneous coronary intervention; STEMI, ST-segment elevation myocardial infarction; NSTEMI, non-STEMI.

^a^ Other races included unknown, multiracial, invalid, and self-identified other.

**Figure 1.**
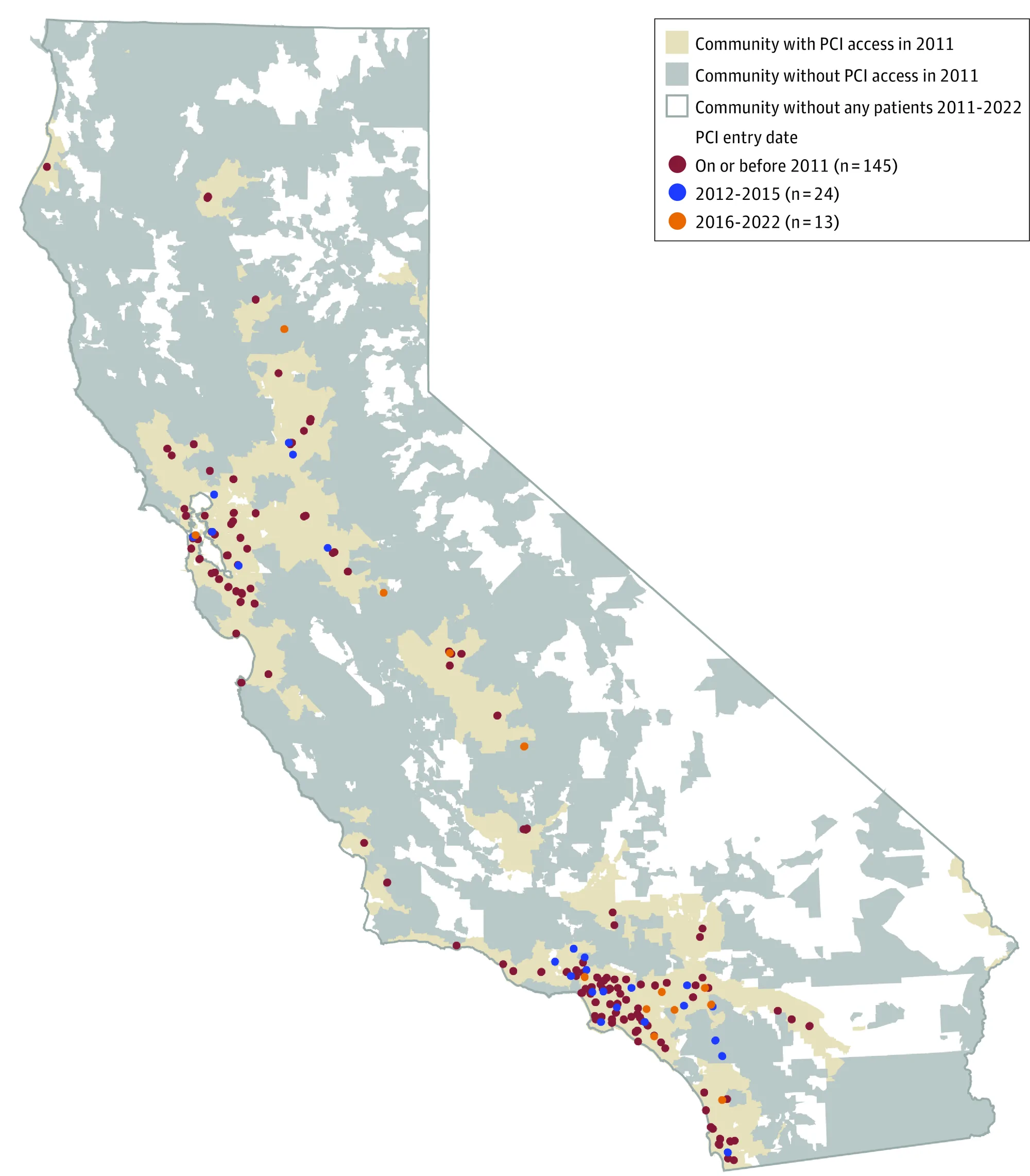
Geographic Distribution of Communities by Baseline Access, Opening Year, and Location of Percutaneous Coronary Intervention (PCI) Facilities in California Between 2011 and 2022

Racial and ethnic composition varied substantially: in communities without baseline PCI access, 2502 were Asian (3%), 3038 Black (3%), 18 002 Hispanic (20%), 60 995 White (68%), and 4184 other races (5%); whereas in communities with baseline PCI access, 60958 were Asian (12%), 44 950 Black (9%), 111 135 Hispanic (22%), 254 331 White (51%), and 28 022 other races (6%). Age distributions were similar between both groups. The prevalence of STEMI (23% [20 541 of 89 504] vs 24% [118 062 of 500 778]) and NSTEMI (77% [68 963 of 89 504] vs 76% [382 716 of 500 778]) was comparable across groups without and with baseline PCI access. Most clinical characteristics were also similar, except for lower rates of vascular disease (10% [9092 of 89 504] vs 15% [75 712 of 500 778]), chronic kidney disease (22% [19 583 of 89 504] vs 29% [147 603 of 500 778]), and kidney failure (21% [18 739 of 89 504] vs 28% [139 180 of 500 778]) among patients living in communities without baseline PCI access.

Communities without baseline access had a lower probability of access and treatment and a higher probability of adverse health outcomes throughout the study period (Figure 2). There was a consistent 20-pp gap in the probability of admission to a PCI facility between the 2 community types (57% [3784 of 6672 patients] in communities without baseline vs 77% [30 528 of 39 639 patients] in communities with baseline in 2011) (Figure 2A). By the end of the study period, 13 889 of 39 678 patients with AMI (35%) received PCI during their hospitalization in communities with baseline access, and 2192 of 7219 (30%) in communities without baseline access (Figure 2B). The probability of unscheduled readmission or ED visit within 30 days initially started at 30% for both community types (1997 of 6672 in communities without baseline access; 11 707 of 39 639 in communities with baseline access), with a slight increase in the gap by 2022 (2303 of 7219 [32%] in communities without baseline access vs 11 466 of 39 678 [29%]) (Figure 2C). Vital statistics are only available through 2019, and the trends in both 30-day and 1-year mortality were similar between the 2 community types (Figure 2D and E). Our multivariate regressions then investigated whether the opening of PCI facilities near a patient’s community were associated with improved access, treatment, and health outcomes separately for these 2 types of communities.

**Figure 2.**
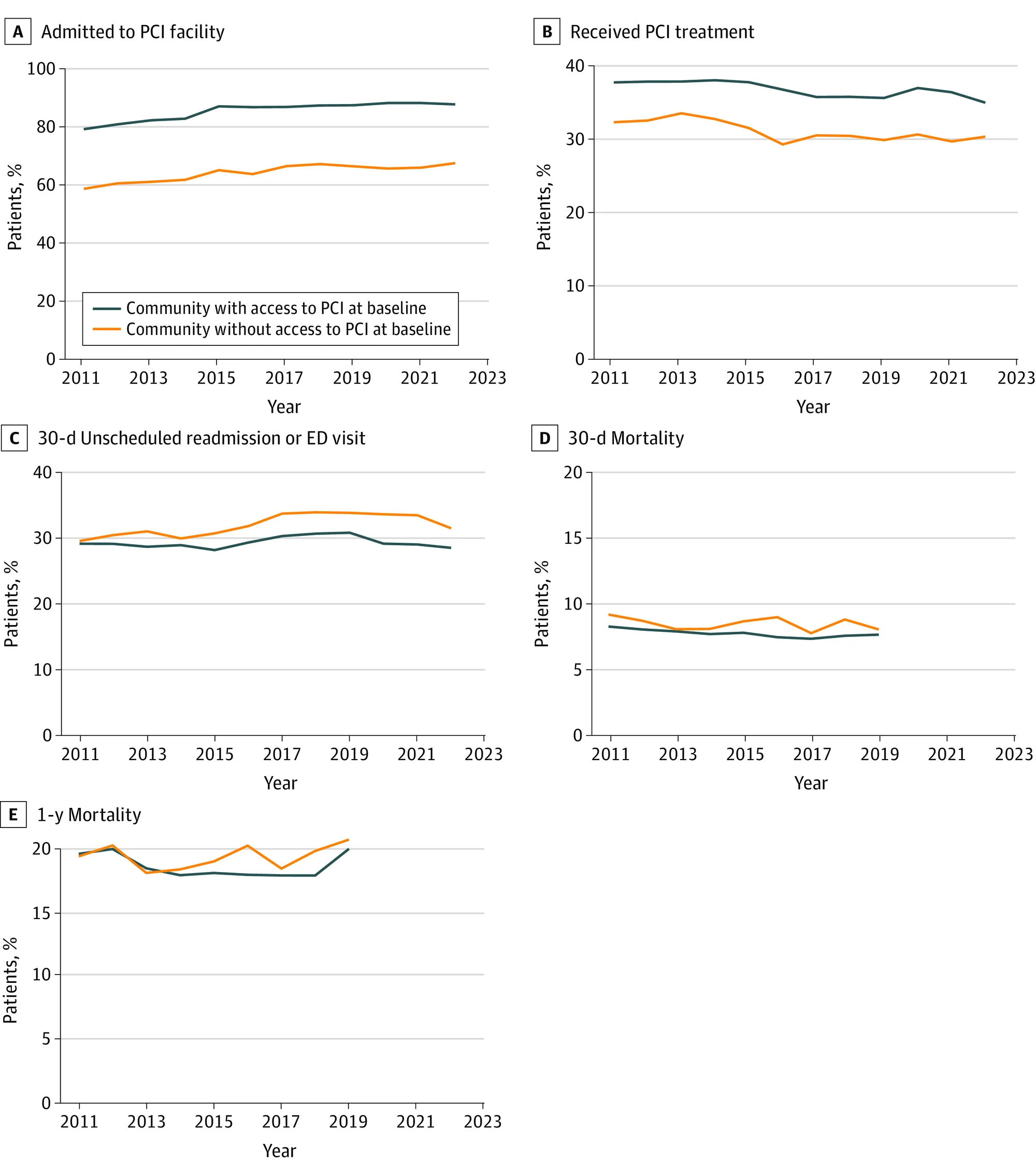
Line Graphs of Patient Outcome Trends Between 2011 and 2022 by Baseline Access The time trends are statistically significantly different between the 2 types of communities for the following outcomes: admitted to percutaneous coronary intervention (PCI) facility (joint *F* test *P* < .001), received PCI treatment (*P* < .001), 30-day unscheduled readmission or emergency department (ED) visit (*P* = .01).

Following PCI facility openings within a 30-minute drive, patients in communities without baseline access were significantly more likely to be admitted to PCI-capable facilities (30.0 pp; 95% CI, 28.2 to 31.8 pp), while gains were modest in communities with existing access (1.5 pp; 95% CI, 1.1 to 1.9 pp) (Figure 3; eTable 2 in Supplement 1). When accounting for baseline rates of access, these represent a 53% relative increase in access to a PCI facility for AMI patients following a PCI opening in communities without baseline access, compared with a 2% relative increase for AMI patients in communities with baseline access. Rates of PCI during the index hospitalization also increased, by 1.0 pp (95% CI, 0.5 to 1.6 pp; equivalent to a 3% relative increase) in communities with baseline access and 5.1 pp (95% CI, 3.3 to 7.0 pp; equivalent to a 16% relative increase) in those without (Figure 3B). Facility openings were associated with a reduction in 30-day unscheduled readmission or ED visit in communities with baseline PCI access (−0.7 pp; 95% CI, −1.2 to −0.2 pp; equivalent to a 2% relative decrease), whereas no significant change was observed in communities without baseline access (Figure 3C). Changes in mortality differed by baseline PCI access. Thirty-day and 1-year mortality improved in communities without baseline PCI access (30 days: −1.8 pp; 95% CI, −3.2 to −0.5 pp; 1 year: −2.8 pp; 95% CI, −4.7 to −1.0 pp), representing 20% and 15% relative decreases in 30-day and 1-year mortality, respectively; mortality rates were unchanged in communities with baseline access (Figure 3D and E).

**Figure 3.**
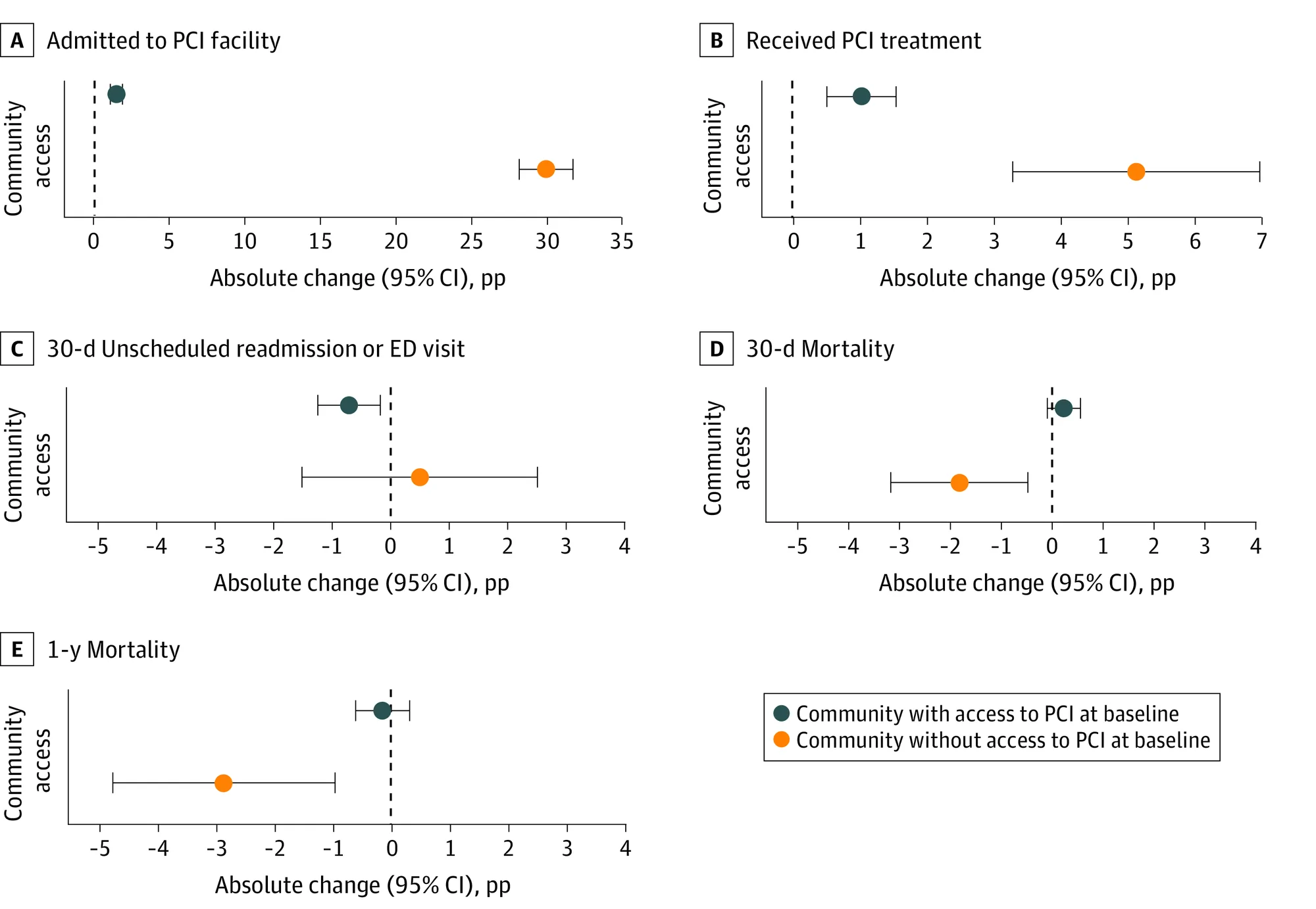
Dot Plots of Changes in Patient Outcomes When a Community Experienced a Percutaneous Coronary Intervention (PCI) Facility Opening Within a 30-Minute Drive Time Based on community fixed effects linear probability models that control for patient demographic and clinical conditions, as well as time trends. Full regression results are available in the online eTable 2 in Supplement 1. ED indicates emergency department; PCI, percutaneous coronary intervention; pp, percentage point.

Finally, we tested whether the association of PCI openings with outcomes differed statistically between the 2 types of communities using a pooled regression that combined communities with and without baseline PCI access but added an interaction term. We found that PCI openings did have a statistically significant difference between the 2 community types across all outcomes, except for 1-year mortality, where the point estimate remained large and negative for the community without baseline access, but was also associated with a large standard error (eTable 3 in Supplement 1).

In our sensitivity analyses, when the exposure was restricted to higher-volume PCI facilities (50 annual cases or more), results remained robust (eTable 4 in Supplement 1). Improvements in 30-day and 1-year survival had larger (30 days: −2.3 pp; 95% CI, −3.8 to −0.8 pp; 1 year: −4.0 pp; 95% CI, −6.3 to −1.7) decreases in mortality, respectively, in communities without baseline access. We also investigated whether the differences in results between the 2 types of communities were due to different trends in patient health profiles over time. Specifically, we looked at the share of patients with chronic conditions (diabetes, vascular disease, heart disease, chronic kidney disease, kidney failure, stroke), prior AMI, or those aged 80 years or older as proxies for medically complex cases. We found that although there were baseline differences, there were no divergent trends in patient health profiles between the 2 community types (eFigure 1 in Supplement 1). We also found that in communities with baseline PCI access, patients with chronic conditions or age 80 years or older had an increased probability of receiving PCI treatment, whereas the association between PCI facility opening and receipt of PCI was similar regardless of patient health profile in communities without baseline access.

In another exploratory analysis, we examined whether trends in the share of patients admitted to low-PCI volume hospitals (defined as facilities with fewer than 200 PCI procedures annually) differed between the 2 community types (eFigure 2 in Supplement 1). We found that for both types of communities, the share of patients admitted to low-volume facilities increased over time, while the share of patients admitted to facilities meeting the minimum volume criteria remained similar.

A final sensitivity analysis focused on patients with STEMI only, because there are clear clinical guidelines and indications for which we would expect no significant change in the likelihood of receiving PCI. Among STEMI patients, in communities without baseline access, there was no significant change in the probability of receiving PCI (eTable 5 in Supplement 1). However, similar to the main analysis, openings were associated with significant reductions in 30-day (−4.8 pp; 95% CI, −7.8 to −1.9 pp) and 1-year (−4.1 pp; 95% CI, −7.7 to −0.5 pp) mortality.

## Discussion

In this statewide cohort study of patients diagnosed with AMI, we found that the opening of new PCI facilities nearby had markedly different outcomes depending on whether communities had baseline PCI access. In communities without access, facility openings substantially increased admission to PCI-capable hospitals, raised the likelihood of receiving PCI, and reduced 30-day and 1-year mortality. In contrast, in communities with existing PCI capacity, additional facility openings produced modest gains in access, small reductions in 30-day unscheduled readmission or ED visits, but no overall improvement in mortality. The baseline PCI rates observed in our study (35% in communities with PCI access and 30% in those without) reflect an all-comer AMI population that includes both patients with STEMI and NSTEMI. Because patients with NSTEMI account for the majority of AMI admissions and may also benefit from PCI expansion, evaluating PCI capacity at the system level is more informative than restricting analyses to STEMI alone.

Our finding of improved mortality outcomes in communities without baseline PCI access is especially important for the rural communities: of the 52 756 patients living in rural communities, 42 584 (81%) lived in communities with no baseline PCI access. The results from the STEMI-only analysis, where there was no change in the receipt of PCI itself, but a significant decrease in 30-day and 1-year mortality, suggest that the survival benefit in previously underserved communities likely operated primarily through more rapid local access to PCI-capable facilities, and thus shorter transfer and ischemic times, rather than through increased PCI use per se for the STEMI population. For patients with NSTEMI, the survival benefit is most likely due to a combination of an increased likelihood of receiving PCI (as shown in Figure 3) and more rapid access.

These results extend previous research on the distribution of cardiac services and their impact on outcomes. Prior work has demonstrated that geographic proximity to revascularization services can meaningfully affect treatment rates and mortality.^19,20^ Our findings build on this research by distinguishing between communities where PCI openings represented true expansion vs those where they might reflect service duplication. The observed benefits in previously underserved areas align with guideline-based expectations that timely PCI improves survival. Conversely, the limited effects in already-served communities suggest a potential duplication of services, although we do see a small reduction in 30-day unscheduled acute care visits, suggesting that PCI capacity could also be a proxy for a greater number of dedicated resources (eg, follow-up clinics, additional services) toward cardiac care.

PCI is a high-reimbursement, high-value service that can bolster hospital revenue, reputation, and competition.^4,21,22^ As a result, many hospitals have invested in the technology, infrastructure, and personnel required to launch PCI programs, even in areas that already have access.^9,23^ In addition, increased PCI capacity may influence treatment patterns by lowering the threshold for intervention. While expanded access may improve care for patients who previously lacked timely treatment, it may also increase use among patients with lower clinical risk. Although our study does not directly evaluate the appropriateness of PCI, these dynamics are important considerations when interpreting the differential effects of expansion across communities. Understanding whether benefits vary by baseline PCI access can inform whether new facilities should prioritize underserved communities or whether additional capacity in already well-served areas is justified.

The divergence in outcomes by baseline access highlights the importance of understanding the surrounding communities’ characteristics in evaluating health system expansion. Where PCI facilities fill genuine geographic gaps, they appear to deliver meaningful improvements in access and survival. On the other hand, openings in markets already served by PCI facilities may lead to potential resource waste, and can potentially fragment patient volumes, especially across hospitals that have just entered the PCI market, leading to lower procedural experience. We do observe that in both community types, the share of patients admitted to facilities that met the annual PCI volume threshold (200 procedures or more) stayed steady over the study period (65% in communities with baseline capacity and 50% without), whereas the share of patients admitted to low-volume PCI hospitals increased over time. In other words, in our study, the introduction of a PCI-capable facility did not appear to siphon patients from existing high-volume hospitals. It is also consistent with prior findings that the introduction of PCI-capable facilities was associated with a statistically significant increase in procedure volume measured at the community level by 8% and 20% in communities with and without baseline access to PCI, respectively,^24^ suggesting that some of the additional capacity is a response to unmet patient needs in those communities.

Our results have implications for health care planning and policy. Because hospitals’ decisions to open PCI programs are often driven by market demand, procedural volume, and return on investment rather than population outcomes, alignment with community need is unlikely to occur through market forces alone, making these findings most actionable for regulatory policy bodies. While California had abolished the practice of issuing certificate of need since the 1980s, local public health agencies’ decisions to incentivize new PCI programs should consider not only clinical guidelines but also the baseline distribution of services. Policymakers and regulators may therefore wish to incorporate community-level need and procedural volume thresholds into facility planning processes.

### Limitations

This study had several important limitations. First, we used administrative data, which may introduce misclassification, and a lack of detailed clinical data, such as infarct size, ischemic time, or appropriateness of PCI. However, we do not expect these data errors to be systematically different between patients or across communities. Second, there are measurement errors in our volume-based PCI-capable hospital designation as well as in determining exposure to PCI capacity for a given community using drive time, where when queried for traffic time, we set the parameters such that they do not account for traffic flow and other time-dependent effects. Such errors will lead to attenuation bias, which makes our estimates more conservative. Third, using California data enabled us to capture all payers, not just Medicare, and all settings of patient encounters. While California comprises 12% of the US population and has diverse backgrounds, our results may not be generalizable to the rest of the nation, particularly to states with different policies and geography. Fourth, while we used baseline PCI access as a proxy, we cannot directly ascertain whether the opening of PCI near a community is a response to unmet needs or duplication of services. Finally, our results show association and not causality, as we were unable to assess patient preferences or referral patterns. These unobserved factors might confound the association between PCI opening and patient outcomes.

## Conclusions

In this cohort study of 590 282 AMI hospitalizations in California, the opening of PCI facilities was associated with substantial improvements in access, treatment, and survival in communities without prior PCI availability, but provided limited or mixed benefits in communities that already had PCI capacity. These findings suggest that the expansion of specialized cardiac services within the health system should be targeted to underserved areas to maximize patient benefits and avoid unintended harms associated with service duplication.

## Notes

Supplement 1. eTable 1. *ICD-9*, *ICD-10*, and CPT procedure codes for identifying PCI eTable 2. Complete regression results of figure eTable 3. Pooled regression testing community differences eTable 4. Sensitivity analysis focusing on the opening of PCI facilities with at least 50 PCI volume annually eTable 5. Sensitivity analysis focusing on the STEMI population only eFigure 1. Trends in the share of medically complex patients by baseline PCI access eFigure 2. Trends in the share of patients admitted to hospitals, by baseline PCI access and hospital PCI volume

Supplement 2. Data sharing statement
