# Supplementary material for: Percutaneous Coronary Intervention Facility Access and Clinical Outcomes Among Patients With Acute Myocardial Infarction
Source: JAMA Netw Open. 2026 Oct 12;9(10):e2638687. doi: 10.1001/jamanetworkopen.2026.38687 (PMC13659571; doi:10.1001/jamanetworkopen.2026.38687)
Supplement: Supplementary file 1 [file jamanetwopen-e2638687-s001.pdf]

Supplemental Online Content

Shen Y, Hsia RY. Percutaneous coronary intervention facility access and clinical outcomes among patients with acute myocardial infarction. *JAMA Netw Open.* 2026;9(10):e2638687. doi:10.1001/jamanetworkopen.2026.38687

eTable 1. *ICD-9*, *ICD-10*, and CPT procedure codes for identifying PCI

eTable 2. Complete regression results of figure

eTable 3. Pooled regression testing community differences

eTable 4. Sensitivity analysis focusing on the opening of PCI facilities with at least 50 PCI volume annually

eTable 5. Sensitivity analysis focusing on the STEMI population only

eFigure 1. Trends in the share of medically complex patients by baseline PCI access

eFigure 2. Trends in the share of patients admitted to hospitals, by baseline PCI access and hospital PCI volume

This supplemental material has been provided by the authors to give readers additional information about their work.

© 2026 Shen Y et al. *JAMA Network Open*.

eTable 1. ICD-9, ICD-10, and CPT Procedure Codes for Identifying PCI

ICD-9 Code	ICD-10 Code	CPT
0066 3601 3602 3605 3606 3607	02703xx 02704xx 02713xx 02714xx 02723xx 02724xx 02733xx 02734xx 02C03Z6 02C03ZZ 02C04Z6 02C04ZZ 02C13Z6 02C13ZZ 02C14Z6 02C14ZZ 02C23Z6 02C23ZZ 02C24Z6 02C24ZZ 02C33Z6 02C33ZZ 02C34Z6 02C34ZZ 02H03DZ 02H03YZ 02HI3DZ 02H13YZ 02H23DZ 02H23YZ 02H33DZ 02H33YZ X2C0361 X2C1361 X2C2361 X2C3361	92920 92921 92924 92925 92928 92929 92933 92934 92937 92938 92941 92943 92944 92973 92975 92978 92979 93571 93572 92980 92981 92982 92984 92995 92996

ICD = International Classification of Diseases; CPT = Current Procedural Terminology

© 2026 Shen Y et al. *JAMA Network Open*.

eTable 2. Complete Regression Results of Figure 2 for Communities

A. With Baseline Access to PCI Within 30 Minutes

	**Admitted to PCI facility**	**Received PCI during care episode**	**Unplanned visit within 30 days**	**30-day mortality**	**1-year mortality**
Baseline mean	77%	38%	30%	8%	20%
On & after yr of newly opened PCI lab within 30mins	1.48**	1.04**	-0.71**	0.23	-0.14
	[1.08,1.89]	[0.52,1.55]	[-1.24,-0.18]	[-0.09,0.56]	[-0.60,0.32]
Female	-2.02**	-5.65**	1.42**	-0.19*	-0.72**
	[-2.23,-1.81]	[-5.91,-5.38]	[1.15,1.70]	[-0.37,-0.01]	[-0.98,-0.47]
African-American	-3.75**	-7.43**	3.86**	-1.30**	-0.71**
	[-4.16,-3.35]	[-7.94,-6.92]	[3.33,4.39]	[-1.65,-0.96]	[-1.20,-0.21]
Hispanic	-1.09**	-3.21**	0.49**	-0.84**	-1.87**
	[-1.37,-0.81]	[-3.57,-2.85]	[0.12,0.86]	[-1.08,-0.60]	[-2.21,-1.52]
Asian or Pacific Islander	-1.30**	-2.82**	-1.33**	-0.90**	-2.20**
	[-1.64,-0.96]	[-3.25,-2.39]	[-1.78,-0.89]	[-1.19,-0.61]	[-2.62,-1.78]
Other races	3.84**	3.02**	-1.76**	-0.67**	-1.35**
	[3.37,4.31]	[2.42,3.61]	[-2.37,-1.14]	[-1.09,-0.26]	[-1.96,-0.74]
age 60-64	0.63**	-0.01	-0.97**	0.77**	1.51**
	[0.30,0.97]	[-0.43,0.41]	[-1.41,-0.54]	[0.49,1.06]	[1.10,1.92]
age 65-69	1.07**	-0.17	-1.77**	1.31**	1.85**
	[0.69,1.44]	[-0.65,0.31]	[-2.27,-1.28]	[0.98,1.63]	[1.38,2.31]
age 70-74	0.77**	-1.30**	-1.76**	2.16**	4.02**
	[0.37,1.17]	[-1.81,-0.79]	[-2.29,-1.23]	[1.81,2.50]	[3.52,4.51]
age 75-79	0.36	-2.50**	-1.40**	3.42**	6.53**
	[-0.05,0.78]	[-3.03,-1.97]	[-1.95,-0.85]	[3.06,3.78]	[6.02,7.04]
age 80-84	-0.34	-4.20**	-1.94**	5.61**	11.31**
	[-0.78,0.10]	[-4.76,-3.64]	[-2.52,-1.36]	[5.23,5.98]	[10.77,11.84]
age 85-99	-2.65**	-12.76**	-4.06**	11.93**	22.63**
	[-3.08,-2.23]	[-13.30,-12.21]	[-4.62,-3.50]	[11.57,12.29]	[22.11,23.14]
Medicare	-0.38*	-0.90**	2.79**	0.68**	2.35**
	[-0.70,-0.06]	[-1.31,-0.50]	[2.37,3.21]	[0.40,0.95]	[1.96,2.74]
Medicaid	-1.13**	-0.99**	2.57**	0.24	0.86**
	[-1.47,-0.78]	[-1.43,-0.55]	[2.11,3.02]	[-0.06,0.54]	[0.43,1.29]
Indigent care	10.20**	10.37**	-2.32**	-1.40**	-2.80**
	[9.06,11.35]	[8.91,11.83]	[-3.83,-0.81]	[-2.27,-0.52]	[-4.01,-1.60]
Privately insured	-3.27**	-1.60**	-1.38**	1.49**	1.44**
	[-3.95,-2.59]	[-2.46,-0.73]	[-2.28,-0.48]	[0.94,2.05]	[0.66,2.23]
Other insurance	3.61**	2.86**	-4.03**	0.46	0.88
	[2.86,4.36]	[1.91,3.81]	[-5.01,-3.05]	[-0.19,1.10]	[-0.05,1.80]
STEMI	9.72**	35.56**	-2.80**	4.02**	2.10**
	[9.48,9.95]	[35.26,35.86]	[-3.11,-2.50]	[3.82,4.22]	[1.81,2.38]

© 2026 Shen Y et al. *JAMA Network Open*.

Had CABG or AMI in prior yr	7.34**	-1.58**	1.50**	0.02	3.09**
	[7.09,7.60]	[-1.90,-1.25]	[1.17,1.84]	[-0.20,0.24]	[2.77,3.40]
Pulmonary Circulation disorders	0.67*	-4.40**	-0.57	1.80**	4.78**
	[0.12,1.22]	[-5.10,-3.70]	[-1.30,0.15]	[1.37,2.23]	[4.19,5.37]
Liver disease	-0.68*	-2.34**	2.29**	1.64**	4.17**
	[-1.24,-0.12]	[-3.05,-1.63]	[1.56,3.03]	[1.13,2.14]	[3.44,4.90]
Cancer	0.26	-5.70**	2.29**	9.04**	22.70**
	[-0.31,0.84]	[-6.44,-4.97]	[1.53,3.05]	[8.54,9.53]	[22.00,23.41]
Dementia	-1.10**	-7.39**	-3.11**	4.29**	9.49**
	[-1.59,-0.61]	[-8.01,-6.77]	[-3.75,-2.47]	[3.85,4.73]	[8.85,10.14]
Valvular disease	3.63**	-2.95**	0.54**	0.01	1.87**
	[3.32,3.94]	[-3.34,-2.56]	[0.13,0.94]	[-0.25,0.28]	[1.50,2.25]
Hypertension	5.04**	5.52**	-0.99**	-3.18**	-4.32**
	[4.79,5.29]	[5.20,5.85]	[-1.33,-0.66]	[-3.40,-2.97]	[-4.63,-4.01]
Chronic pulmonary disease	-0.69**	-2.62**	2.48**	0.11	2.94**
	[-0.94,-0.44]	[-2.95,-2.30]	[2.15,2.82]	[-0.11,0.32]	[2.63,3.25]
Rheumatoid arthritis/collagen vascular	1.88**	1.98**	0.78	-0.51	0.85*
	[1.23,2.53]	[1.15,2.81]	[-0.08,1.64]	[-1.07,0.04]	[0.06,1.64]
Coagulation deficiency	5.30**	-7.37**	-4.04**	4.50**	4.52**
	[4.92,5.69]	[-7.86,-6.88]	[-4.55,-3.53]	[4.16,4.83]	[4.04,5.00]
Obesity	2.26**	2.02**	-1.65**	-1.08**	-3.47**
	[2.00,2.52]	[1.69,2.35]	[-1.99,-1.31]	[-1.30,-0.85]	[-3.80,-3.15]
Substance abuse	1.31**	-3.78**	3.01**	-1.15**	-1.29**
	[0.91,1.70]	[-4.28,-3.28]	[2.49,3.53]	[-1.49,-0.81]	[-1.78,-0.80]
Depression	4.46**	1.87**	-1.19**	-0.57**	0.21
	[3.97,4.94]	[1.25,2.49]	[-1.83,-0.55]	[-0.96,-0.18]	[-0.34,0.76]
Psychosis	-4.05**	-3.57**	3.14**	-0.47*	0.31
	[-4.48,-3.62]	[-4.12,-3.03]	[2.57,3.70]	[-0.83,-0.10]	[-0.21,0.83]
Hypothyroidism	1.14**	0.74**	0.18	-1.28**	-1.06**
	[0.83,1.45]	[0.35,1.14]	[-0.23,0.58]	[-1.55,-1.02]	[-1.44,-0.68]
Paralysis and neurological disorder	-1.49**	-4.30**	0.93**	4.76**	7.87**
	[-1.87,-1.11]	[-4.78,-3.81]	[0.43,1.43]	[4.43,5.08]	[7.41,8.34]
Chronic Peptic ulcer disease	2.45**	-1.47	-1.36	-1.07	0.46
	[1.13,3.76]	[-3.14,0.21]	[-3.09,0.37]	[-2.30,0.16]	[-1.38,2.29]
Weight loss	-5.61**	-6.42**	-1.60**	5.18**	12.60**
	[-6.11,-5.12]	[-7.05,-5.79]	[-2.25,-0.95]	[4.76,5.60]	[11.99,13.20]
Fluid and electrolyte disorders	2.69**	-2.35**	-2.02**	6.00**	7.25**
	[2.45,2.93]	[-2.65,-2.04]	[-2.33,-1.70]	[5.79,6.20]	[6.95,7.54]

© 2026 Shen Y et al. *JAMA Network Open*.

Anemia (blood loss and deficiency)	1.24**	-0.81**	2.28**	-0.77**	3.14**
	[0.98,1.50]	[-1.14,-0.48]	[1.94,2.62]	[-0.99,-0.55]	[2.83,3.46]
Stroke	3.24**	-3.29**	-2.12**	2.47**	3.07**
	[2.77,3.72]	[-3.90,-2.68]	[-2.74,-1.49]	[2.07,2.88]	[2.49,3.66]
Chronic Kidney Disease	1.01**	-0.01	2.20**	-2.82**	-2.56**
	[0.37,1.66]	[-0.83,0.82]	[1.35,3.05]	[-3.65,-1.98]	[-3.79,-1.33]
Heart failure	1.70**	-3.46**	1.73**	4.77**	10.63**
	[1.49,1.92]	[-3.74,-3.18]	[1.44,2.02]	[4.58,4.96]	[10.36,10.90]
Diabetes	0.31**	0.36**	2.39**	0.25**	1.56**
	[0.10,0.52]	[0.09,0.63]	[2.11,2.66]	[0.07,0.43]	[1.30,1.82]
Renal failure	-2.40**	-3.06**	2.45**	5.30**	10.13**
	[-3.05,-1.75]	[-3.89,-2.24]	[1.59,3.30]	[4.46,6.13]	[8.90,11.36]
Peripheral vascular disease	-2.69**	-2.33**	2.72**	-0.29*	0.75**
	[-2.97,-2.41]	[-2.68,-1.97]	[2.35,3.09]	[-0.53,-0.05]	[0.41,1.09]
Reference year: 2011					
2012	1.04**	0.14	0.12	-0.29	0.39
	[0.54,1.54]	[-0.49,0.78]	[-0.54,0.78]	[-0.66,0.09]	[-0.12,0.91]
2013	2.62**	0.64	-0.43	-0.40*	-1.15**
	[2.11,3.12]	[-0.01,1.28]	[-1.10,0.24]	[-0.78,-0.02]	[-1.68,-0.63]
2014	3.28**	1.11**	-0.39	-0.46*	-1.36**
	[2.75,3.81]	[0.43,1.79]	[-1.09,0.31]	[-0.87,-0.06]	[-1.91,-0.80]
2015	7.23**	1.67**	-1.26**	-0.52*	-1.28**
	[6.64,7.82]	[0.92,2.43]	[-2.04,-0.49]	[-0.97,-0.07]	[-1.90,-0.65]
2016	7.19**	2.31**	-0.78	-0.98**	-1.28**
	[6.26,8.11]	[1.13,3.49]	[-2.00,0.44]	[-1.68,-0.28]	[-2.23,-0.32]
2017	7.11**	1.98**	0.30	-1.20**	-1.59**
	[6.19,8.03]	[0.80,3.16]	[-0.92,1.52]	[-1.90,-0.50]	[-2.55,-0.64]
2018	7.71**	2.46**	0.46	-0.93**	-1.62**
	[6.78,8.64]	[1.28,3.64]	[-0.76,1.68]	[-1.63,-0.23]	[-2.58,-0.66]
2019	7.61**	1.99**	0.71	-0.82*	0.18
	[6.68,8.54]	[0.81,3.18]	[-0.51,1.94]	[-1.53,-0.12]	[-1.20,1.56]
2020	8.33**	2.66**	-0.84		
	[7.39,9.27]	[1.46,3.86]	[-2.08,0.40]		
2021	8.21**	2.35**	-0.83		
	[7.27,9.15]	[1.15,3.55]	[-2.07,0.41]		
2022	7.93**	1.15	-1.23		
	[6.99,8.87]	[-0.05,2.35]	[-2.47,0.01]		
On and after switch to ICD-10 (Oct 1, 2015)	-0.73	-3.55**	1.07*	-0.18	-0.71
	[-1.47,0.01]	[-4.49,-2.61]	[0.09,2.04]	[-0.74,0.37]	[-1.47,0.04]
Constant term	69.29**	37.11**	26.37**	1.94**	3.45**
	[68.84,69.75]	[36.53,37.70]	[25.77,26.98]	[1.57,2.30]	[2.94,3.96]
N	499,786	500,739	500,739	380,341	343,632

© 2026 Shen Y et al. *JAMA Network Open*.

B. Without Baseline Access to PCI Within 30 Minutes

	**Admitted to PCI facility**	**Received PCI during care episode**	**Unplanned visit within 30 days**	**30-day mortality**	**1-year mortality**
Baseline mean	57%	32%	30%	9%	19%
On & after yr of newly opened PCI lab within 30mins	30.01**	5.12**	0.49	-1.82**	-2.84**
	[28.23,31.80]	[3.29,6.96]	[-1.51,2.50]	[-3.17,-0.48]	[-4.74,-0.95]
Female	-6.02**	-5.68**	2.61**	-0.37	-0.63*
	[-6.60,-5.44]	[-6.28,-5.08]	[1.96,3.27]	[-0.81,0.07]	[-1.25,-0.01]
African-American	-5.64**	-7.62**	4.55**	-0.93	0.08
	[-7.21,-4.06]	[-9.24,-6.00]	[2.78,6.31]	[-2.12,0.26]	[-1.61,1.77]
Hispanic	-1.98**	-3.49**	0.88	-1.30**	-2.20**
	[-2.80,-1.17]	[-4.33,-2.65]	[-0.04,1.79]	[-1.92,-0.68]	[-3.07,-1.32]
Asian or Pacific Islander	-0.30	-2.48**	-1.12	-0.38	-1.78
	[-2.01,1.40]	[-4.23,-0.72]	[-3.04,0.79]	[-1.69,0.94]	[-3.63,0.06]
Other races	8.18**	5.73**	-1.77*	-1.51*	-2.55**
	[6.62,9.73]	[4.13,7.32]	[-3.51,-0.02]	[-2.75,-0.26]	[-4.36,-0.74]
age 60-64	0.38	0.33	-1.42**	1.05**	1.63**
	[-0.56,1.31]	[-0.64,1.29]	[-2.48,-0.37]	[0.35,1.76]	[0.63,2.62]
age 65-69	-0.86	-2.29**	-2.41**	1.49**	2.13**
	[-1.94,0.22]	[-3.40,-1.17]	[-3.63,-1.20]	[0.68,2.31]	[0.98,3.27]
age 70-74	-2.58**	-2.77**	-1.73**	3.10**	5.37**
	[-3.70,-1.46]	[-3.93,-1.62]	[-2.99,-0.46]	[2.25,3.96]	[4.17,6.57]
age 75-79	-3.80**	-4.09**	-1.37*	4.50**	8.96**
	[-4.98,-2.63]	[-5.31,-2.88]	[-2.70,-0.05]	[3.61,5.40]	[7.70,10.22]
age 80-84	-6.67**	-5.79**	-0.63	7.64**	14.10**
	[-7.92,-5.43]	[-7.08,-4.51]	[-2.03,0.77]	[6.70,8.57]	[12.79,15.41]
age 85-99	-13.43**	-11.62**	-4.34**	13.56**	24.07**
	[-14.66,-12.20]	[-12.89,-10.36]	[-5.72,-2.96]	[12.64,14.48]	[22.78,25.36]
Medicare	-0.60	-0.50	5.23**	0.68	3.30**
	[-1.53,0.33]	[-1.46,0.45]	[4.19,6.28]	[-0.02,1.39]	[2.31,4.29]
Medicaid	-4.14**	-3.61**	5.98**	0.00	1.44**
	[-5.15,-3.13]	[-4.65,-2.56]	[4.85,7.12]	[-0.77,0.78]	[0.34,2.53]
Indigent care	13.01**	10.96**	2.10	-2.01	-2.52
	[9.52,16.49]	[7.38,14.55]	[-1.81,6.01]	[-4.34,0.31]	[-5.66,0.63]
Privately insured	-5.94**	-2.79**	1.03	1.66*	1.69
	[-7.85,-4.03]	[-4.75,-0.82]	[-1.12,3.17]	[0.28,3.05]	[-0.24,3.62]
Other insurance	3.50**	1.91*	0.17	-0.86	-0.04
	[1.74,5.26]	[0.10,3.72]	[-1.81,2.15]	[-2.23,0.50]	[-1.96,1.89]
STEMI	19.55**	33.93**	-7.01**	3.81**	1.59**
	[18.88,20.22]	[33.25,34.62]	[-7.76,-6.26]	[3.31,4.31]	[0.88,2.29]
	17.77**	3.34**	-1.37**	-0.38	2.26**

© 2026 Shen Y et al. *JAMA Network Open*.

Had CABG or AMI in prior year	[17.06,18.48]	[2.61,4.07]	[-2.17,-0.57]	[-0.92,0.16]	[1.50,3.02]
Pulmonary Circulation disorders	1.67*	-3.42**	-1.32	2.44**	3.63**
	[0.07,3.28]	[-5.08,-1.77]	[-3.12,0.49]	[1.34,3.54]	[2.14,5.12]
Liver disease	0.83	-2.28*	1.93	2.09**	7.24**
	[-0.93,2.60]	[-4.09,-0.46]	[-0.06,3.91]	[0.70,3.47]	[5.26,9.22]
Cancer	-0.52	-2.59**	0.90	9.33**	22.22**
	[-2.27,1.22]	[-4.38,-0.79]	[-1.06,2.87]	[8.00,10.65]	[20.37,24.07]
Dementia	-0.32	-5.08**	-5.48**	4.16**	9.14**
	[-1.84,1.20]	[-6.65,-3.52]	[-7.19,-3.77]	[2.96,5.36]	[7.41,10.86]
Valvular disease	12.34**	0.82	-2.17**	-0.47	-0.76
	[11.41,13.27]	[-0.13,1.78]	[-3.22,-1.13]	[-1.17,0.23]	[-1.75,0.23]
Hypertension	12.60**	8.54**	-2.14**	-3.63**	-5.34**
	[11.94,13.27]	[7.86,9.23]	[-2.89,-1.40]	[-4.13,-3.13]	[-6.04,-4.64]
Chronic pulmonary disease	-0.10	-2.44**	1.99**	0.55*	3.59**
	[-0.80,0.60]	[-3.17,-1.72]	[1.20,2.78]	[0.03,1.08]	[2.85,4.32]
Rheumatoid arthritis/collagen vascular	3.66**	2.21*	1.62	0.08	2.44*
	[1.72,5.61]	[0.21,4.21]	[-0.57,3.81]	[-1.35,1.52]	[0.43,4.45]
Coagulation deficiency	11.56**	-4.58**	-5.43**	3.53**	2.79**
	[10.28,12.84]	[-5.90,-3.27]	[-6.87,-3.99]	[2.56,4.51]	[1.42,4.16]
Obesity	10.68**	6.72**	-3.87**	-2.19**	-4.20**
	[9.91,11.46]	[5.93,7.52]	[-4.75,-3.00]	[-2.78,-1.60]	[-5.03,-3.37]
Substance abuse	6.20**	0.21	1.96**	-1.19**	-1.69**
	[5.11,7.29]	[-0.91,1.33]	[0.74,3.19]	[-2.03,-0.36]	[-2.88,-0.51]
Depression	2.08**	1.82*	-1.28	-1.49**	0.54
	[0.61,3.54]	[0.31,3.33]	[-2.93,0.36]	[-2.51,-0.47]	[-0.86,1.93]
Psychosis	2.33**	-0.54	3.22**	-0.93	-3.36**
	[0.88,3.77]	[-2.03,0.95]	[1.59,4.84]	[-2.00,0.15]	[-4.87,-1.85]
Hypothyroidism	5.27**	3.13**	-1.28*	-1.29**	-1.78**
	[4.37,6.17]	[2.21,4.06]	[-2.29,-0.27]	[-1.97,-0.62]	[-2.73,-0.82]
Paralysis and other neurological disorder	-2.20**	-3.12**	0.97	5.48**	8.18**
	[-3.35,-1.04]	[-4.30,-1.93]	[-0.32,2.27]	[4.62,6.34]	[6.96,9.39]
Chronic Peptic ulcer disease	9.01**	4.75*	-5.55*	-2.07	3.55
	[4.99,13.02]	[0.62,8.88]	[-10.07,-1.04]	[-5.42,1.28]	[-1.35,8.45]
Weight loss	-1.55	-3.41**	-2.37**	4.83**	11.39**
	[-3.15,0.05]	[-5.05,-1.76]	[-4.17,-0.57]	[3.64,6.03]	[9.70,13.07]
Fluid and electrolyte disorders	4.49**	-1.65**	-3.15**	6.29**	7.86**
	[3.78,5.20]	[-2.39,-0.92]	[-3.95,-2.34]	[5.74,6.83]	[7.09,8.63]
Anemia (blood loss and deficiency)	4.22**	0.22	2.26**	-0.36	3.61**
	[3.39,5.06]	[-0.64,1.07]	[1.32,3.19]	[-0.99,0.26]	[2.74,4.49]
Stroke	9.59**	-2.78**	-5.20**	4.28**	4.98**
	[8.10,11.08]	[-4.31,-1.24]	[-6.88,-3.52]	[3.17,5.40]	[3.41,6.55]
	10.15**	3.32**	2.04	-4.43**	-3.32*

© 2026 Shen Y et al. *JAMA Network Open*.

Chronic Kidney Disease	[8.09,12.20]	[1.21,5.44]	[-0.27,4.35]	[-6.62,-2.23]	[-6.43,-0.22]
Heart failure	3.13**	-1.94**	1.36**	5.37**	11.10**
	[2.50,3.77]	[-2.59,-1.29]	[0.65,2.07]	[4.89,5.85]	[10.42,11.78]
Diabetes	0.56	0.33	2.12**	0.17	1.90**
	[-0.05,1.17]	[-0.30,0.96]	[1.43,2.80]	[-0.29,0.63]	[1.25,2.55]
Renal failure	-10.66**	-5.22**	1.58	6.34**	11.68**
	[-12.73,-8.60]	[-7.35,-3.10]	[-0.74,3.90]	[4.15,8.53]	[8.59,14.78]
Peripheral vascular disease	5.93**	-0.72	1.02	0.22	2.40**
	[4.99,6.86]	[-1.68,0.24]	[-0.03,2.08]	[-0.47,0.92]	[1.44,3.37]
Reference year: 2011					
2012	-1.44*	-0.01	0.56	-0.59	0.37
	[-2.85,-0.03]	[-1.46,1.44]	[-1.02,2.15]	[-1.52,0.33]	[-0.86,1.61]
2013	-1.21	1.31	1.16	-0.94*	-1.39*
	[-2.63,0.21]	[-0.15,2.77]	[-0.44,2.75]	[-1.87,-0.01]	[-2.63,-0.15]
2014	0.34	1.86*	-0.29	-0.94*	-1.43*
	[-1.07,1.75]	[0.41,3.31]	[-1.88,1.29]	[-1.86,-0.01]	[-2.67,-0.20]
2015	2.42**	1.13	0.38	-0.05	-0.08
	[0.92,3.91]	[-0.41,2.66]	[-1.30,2.06]	[-1.03,0.94]	[-1.40,1.23]
2016	0.94	-0.58	1.84	0.29	2.23*
	[-1.58,3.46]	[-3.18,2.01]	[-0.99,4.68]	[-1.36,1.94]	[0.02,4.44]
2017	1.70	0.39	3.76**	-0.47	1.20
	[-0.83,4.23]	[-2.21,2.99]	[0.91,6.60]	[-2.12,1.19]	[-1.01,3.42]
2018	2.78*	1.29	3.80**	0.48	2.22*
	[0.25,5.31]	[-1.32,3.90]	[0.95,6.65]	[-1.18,2.14]	[0.00,4.44]
2019	2.23	0.57	3.68*	-0.10	3.17
	[-0.30,4.76]	[-2.04,3.17]	[0.83,6.52]	[-1.77,1.56]	[-0.08,6.42]
2020	1.61	1.15	3.59*		
	[-0.94,4.16]	[-1.48,3.78]	[0.72,6.46]		
2021	1.07	-0.07	3.68*		
	[-1.49,3.63]	[-2.70,2.56]	[0.80,6.56]		
2022	3.14*	1.07	1.49		
	[0.57,5.71]	[-1.57,3.71]	[-1.39,4.38]		
On and after switch to ICD-10 (Oct 1, 2015)	-1.11	-3.00**	0.09	-0.76	-2.36*
	[-3.23,1.02]	[-5.19,-0.81]	[-2.30,2.48]	[-2.15,0.63]	[-4.22,-0.51]
Constant term	38.62**	24.91**	29.10**	3.33**	4.41**
	[37.31,39.94]	[23.56,26.26]	[27.62,30.58]	[2.42,4.25]	[3.16,5.67]
N	89,310	89,476	89,476	67,106	60,303

© 2026 Shen Y et al. *JAMA Network Open*.

eTable 3. Pooled Regression Testing Community Differences

	Admitted to PCI facility	Received PCI during care episode	Unplanned visit within 30 days	30-day mortality	1-year mortality
Ref group: community with baseline access					
On & after yr of newly opened PCI lab within 30mins	2.09**	1.10**	-1.06**	0.13	-0.40
	[1.70,2.49]	[0.62,1.58]	[-1.56,-0.56]	[-0.18,0.44]	[-0.83,0.04]
Interaction with community without baseline	25.93**	3.80**	2.75**	-1.76**	-1.59
	[24.43,27.43]	[1.97,5.63]	[0.85,4.66]	[-3.02,-0.51]	[-3.38,0.20]
N	589,126	590,246	590,246	447,478	403,961

© 2026 Shen Y et al. *JAMA Network Open*.

eTable 4. Sensitivity Analysis Focusing on the Opening of PCI Facilities with at Least 50 PCI Volume

	**Admitted to PCI facility**	**Received PCI during care episode**	**Unplanned visit within 30 days**	**30-day mortality**	**1-year mortality**
Communities with baseline PCI access					
On & after yr of newly opened PCI lab within 30mins	2.18**	0.23	-0.45	0.10	0.11
	[1.77,2.58]	[-0.28,0.75]	[-0.98,0.08]	[-0.23,0.43]	[-0.35,0.58]
Communities without baseline PCI access					
On & after yr of newly opened PCI lab within 30mins	24.60**	3.87**	0.45	-2.32**	-3.99**
	[22.72,26.48]	[1.94,5.80]	[-1.66,2.56]	[-3.83,-0.81]	[-6.30,-1.68]

© 2026 Shen Y et al. *JAMA Network Open*.

eTable 5. Sensitivity Analysis Focusing on STEMI Patient Population Only

	**Admitted to PCI facility**	**Received PCI during care episode**	**Unplanned visit within 30 days**	**30-day mortality**	**1-year mortality**
Communities with baseline PCI access	90%	71%	25%	8%	14%
On & after yr of newly opened PCI lab within 30mins	0.91** [0.23,1.59]	0.92 [-0.13,1.98]	0.13 [-0.90,1.17]	0.07 [-0.62,0.77]	-0.04 [-0.89,0.80]
# of obs	119,227				
Communities without baseline PCI access	77%	64%	25%	8%	13%
On & after yr of newly opened PCI lab within 30mins	20.23** [16.83,23.63]	-0.84 [-4.94,3.26]	0.73 [-3.17,4.63]	-4.84** [-7.77,-1.91]	-4.08* [-7.71,-0.46]
# of obs	21,133				

© 2026 Shen Y et al. *JAMA Network Open*.

eFigure 1. Trends in the Share of Medically Complex Patients by Baseline PCI Access

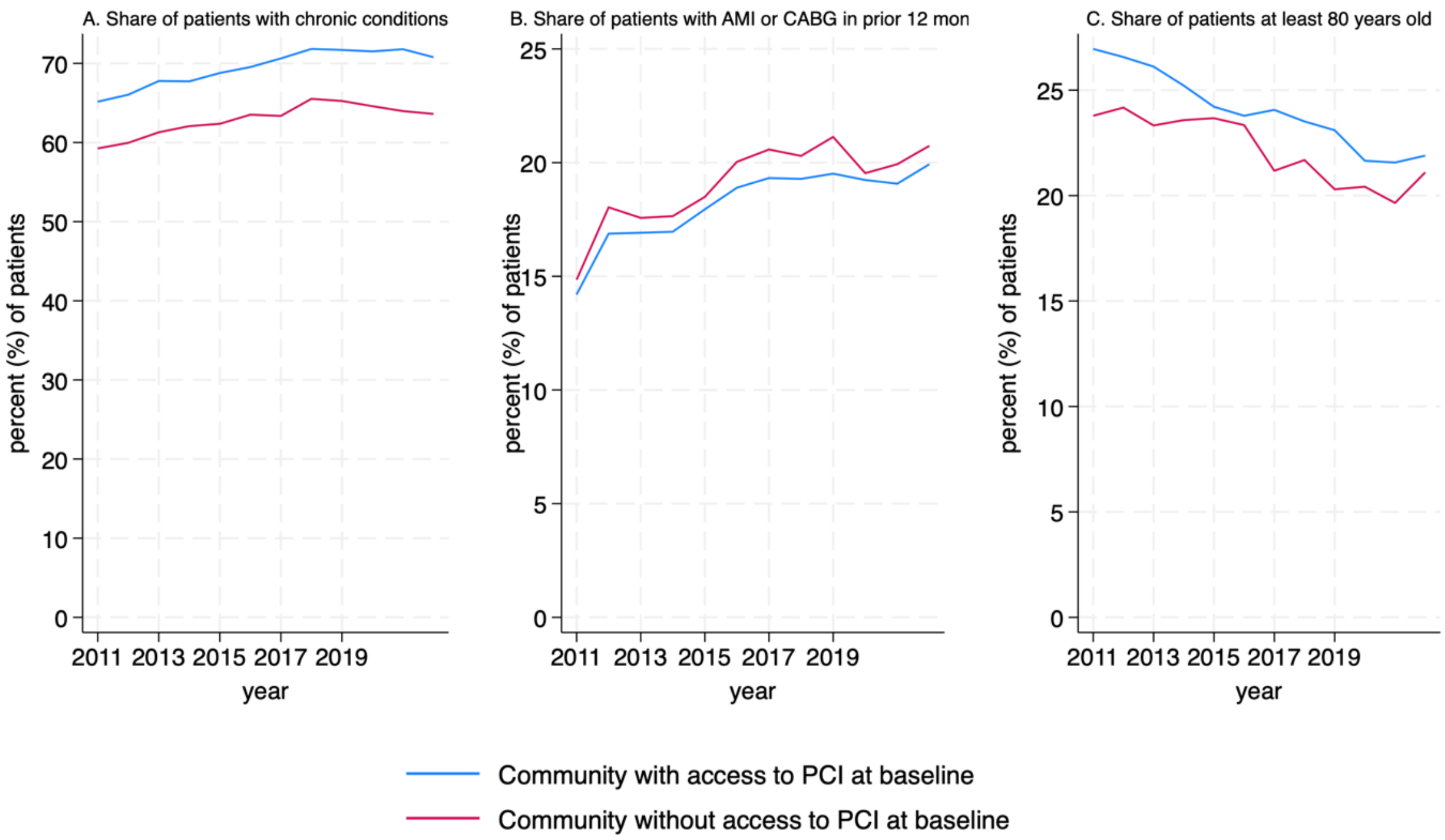

© 2026 Shen Y et al. *JAMA Network Open*.

eFigure 2. Trends in the Share of Patients Admitted to Hospitals by Baseline PCI Access and Hospital PCI Volume

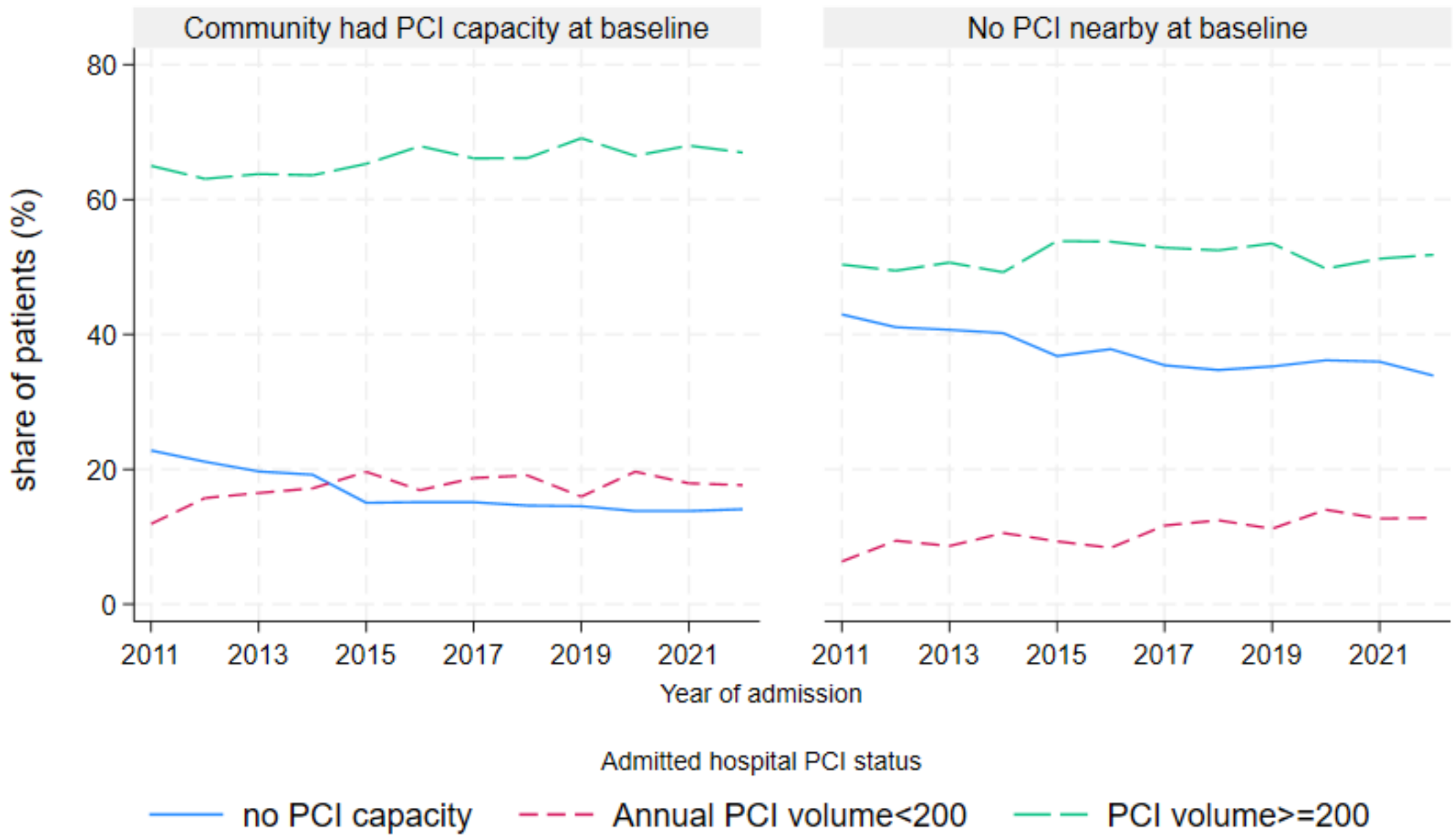

© 2026 Shen Y et al. *JAMA Network Open*.
