# Supplementary material for: Percutaneous Coronary Intervention Facility Access and Clinical Outcomes Among Patients With Acute Myocardial Infarction
Source: JAMA Netw Open. 2026 Oct 12;9(10):e2638687. doi: 10.1001/jamanetworkopen.2026.38687 (PMC13659571; doi:10.1001/jamanetworkopen.2026.38687)
Supplement: Supplementary file 2 [file jamanetwopen-e2638687-s002.pdf]

Data Sharing Statement

Shen. Percutaneous Coronary Intervention Facility Access and Clinical Outcomes Among Patients With Acute Myocardial Infarction. *JAMA Netw Open*. Published October 12, 2026. doi:10.1001/jamanetworkopen.2026.38687

Data
Data available: No
